# Patterns of ICU admissions and outcomes in patients with solid malignancies over the revolution of cancer treatment

**DOI:** 10.1186/s13613-021-00968-5

**Published:** 2021-12-24

**Authors:** Clara Vigneron, Julien Charpentier, Sandrine Valade, Jérôme Alexandre, Samy Chelabi, Lola-Jade Palmieri, Nathalie Franck, Valérie Laurence, Jean-Paul Mira, Matthieu Jamme, Frédéric Pène

**Affiliations:** 1 Service de Médecine Intensive-Réanimation, Hôpital Cochin, Assistance Publique-Hôpitaux de Paris (AP-HP). Centre & Université de Paris, Paris, France; 2 Service de Médecine Intensive-Réanimation, Hôpital Saint-Louis, AP-HP. Nord & Université de Paris, Paris, France; 3 Département d’oncologie Médicale, Hôpital Cochin, AP-HP. Centre & Université de Paris, Paris, France; 4 Service de Pneumologie, Hôpital Cochin, AP-HP. Centre & Université de Paris, Paris, France; 5 Service de Gastro-Entérologie et Oncologie Digestive, Hôpital Cochin, AP-HP. Centre & Université de Paris, Paris, France; 6 Service de Dermatologie-Vénéréologie, Hôpital Cochin, AP-HP. Centre & Université de Paris, Paris, France; 7 Service d’oncologie Médicale, Institut Curie, Paris, France; 8 UMR8104, Institut Cochin, INSERM U1016, CNRS, Université de Paris, Paris, France; 9 Service de Médecine Intensive-Réanimation, Hôpital Privé de l’ouest Parisien, Ramsay Générale de Santé, Trappes, France; 10 INSERM U1018, Centre de Recherche en Epidémiologie et Santé des Populations, Team 5 (EpReC, Renal and Cardiovascular Epidemiology), Université Versailles Saint-Quentin, Villejuif, France

**Keywords:** Solid tumour, ICU, Outcome, Drug-related side effects

## Abstract

**Background:**

Major therapeutic advances including immunotherapy and targeted therapies have been changing the face of oncology and resulted in improved prognosis as well as in new toxic complications. The aim of this study is to appraise the trends in intensive care unit (ICU) admissions and outcomes of critically ill patients with solid malignancies. We performed a retrospective single-centre study over a 12-year period (2007–2018) including adult patients with solid malignancies requiring unplanned ICU admission. Admission patterns were classified as: (i) specific if directly related to the underlying cancer; (ii) non-specific; (iii) drug-related or procedural adverse events.

**Results:**

1525 patients were analysed. Lung and gastro-intestinal tract accounted for the two main tumour sites. The proportion of patients with metastatic diseases increased from 48.6% in 2007–2008 to 60.2% in 2017–2018 (*p* = 0.004). Critical conditions were increasingly related to drug- or procedure-related adverse events, from 8.8% of ICU admissions in 2007–2008 to 16% in 2017–2018 (*p* = 0.01). The crude severity of critical illness at ICU admission did not change over time. The ICU survival rate was 77.4%, without any significant changes over the study period. Among the 1279 patients with complete follow-up, the 1-year survival rate was 33.2%. Independent determinants of ICU mortality were metastatic disease, cancer in progression under treatment, admission for specific complications and the extent of organ failures (invasive and non-invasive ventilation, inotropes/vasopressors, renal replacement therapy and SOFA score). One-year mortality in ICU-survivors was independently associated with lung cancer, metastatic disease, cancer in progression under treatment, admission for specific complications and decision to forgo life-sustaining therapies.

**Conclusion:**

Advances in the management and the prognosis of solid malignancies substantially modified the ICU admission patterns of cancer patients. Despite underlying advanced and often metastatic malignancies, encouraging short-term and long-term outcomes should help changing the dismal perception of critically ill cancer patients.

**Supplementary Information:**

The online version contains supplementary material available at 10.1186/s13613-021-00968-5.

## Background

Oncology represents a striking field of progress in medicine. Continuous improvements in the prognosis of cancer have been ascribed to earlier detection of diseases through extensive population screening, advances in oncologic treatment and improvements in supportive care [1, 2]. Besides the historical paradigm of tumour elimination by surgery, radiotherapy and cytostatic chemotherapy, a better understanding of oncogenic processes and anti-tumoral immunosurveillance led to new therapeutic approaches targeting actionable oncogenic mutations in tumour cells or their immune microenvironment. Once considered refractory, some advanced-staged and metastatic malignancies have thus become liable to targeted therapies or immunotherapy that may allow a longstanding control of the disease and prolonged survival with acceptable quality of life [3]. However, new treatments mean new toxicity profiles and life-threatening side effects requiring intensive care unit (ICU) admission [4–6].

Cancer is definitely a major risk factor of acute critical illness. Patients with malignancies, mostly with solid tumours, account for about one in seven critically ill patients, with a substantial proportion of metastatic diseases (40%) [7]. Hence an international survey reported that 8.8% and 3.3% of patients in the ICU had a history of non-metastatic and metastatic solid cancer, respectively [8]. The recent major changes in the landscape of oncology raise the question of the current indications of ICU admission and the related prognosis in critically ill cancer patients. Although recent studies have well addressed these issues in patients with haematological malignancies, data about patients with solid tumours are scarce, with ICU admission policies based on relatively old studies which do not reflect the current prognosis of cancer.

In the light of the growing incidence of cancer and overall improvement in oncologic prognosis, the aim of this study was to appraise the current trends in unplanned ICU admissions and outcomes of critically ill patients with solid malignancies over a 12-year period.

## Methods

### Patients and setting

We performed a retrospective single-centre study in a 24-bed medical ICU located in a tertiary care hospital with comprehensive oncology departments. Patients admitted for surgical reason are usually hospitalized in a separated surgical ICU. From January 2007 to December 2018, adult patients (age ≥ 18 years) with a diagnosis of solid tumour (known before or during the ICU stay) requiring unplanned ICU admissions were included. Our hospital policy for critically ill oncological patients is based on early ICU admission for the management and the monitoring of organ failures. Non-inclusion criteria were the following: admission to secure a procedure, planned admissions following elective surgery and patients with cancer cured for more than 5 years. For patients with multiple ICU admissions, only the first qualifying ICU stay was considered.

According to French regulations, this study was approved by the ethics committee from the Société de Réanimation de Langue Française (CE SRLF 17–03) which waived the need for signed consent. Some data on the subgroup of patients with lung cancer were previously reported elsewhere [9].

### Data collection

The following data related to the underlying malignancy were collected: date of diagnosis, primary tumour site, cancer staging (localized, advanced, metastatic), oncologic status according to RECIST (newly diagnosed cancer during ICU stay or within 1 month before ICU admission, partial remission including stable disease, complete remission, progression), oncologic treatment within 3 months before ICU admission (surgery, radiotherapy, cytostatic chemotherapy, targeted therapy [i.e. drug targeting a specific gene mutation or protein], immunotherapy). The severity at ICU admission was assessed by the Sequential Organ Failure Assessment (SOFA) score computed from the first 24 h [10]. Organ failure supports were collected throughout the ICU stay, including invasive and non-invasive mechanical ventilation, vasopressors/inotropes and renal replacement therapy. Leukopenia was defined as leukocyte count < 1000/mm^3^. We recorded the decisions to forgo (withholding or withdrawing) life-sustaining therapies (DFLST). The main outcomes were in-ICU survival and one-year survival in ICU survivors with complete follow-up.

### Patterns of ICU admissions

Causes for ICU admissions were classified into three different categories: (i) specific if directly related to the underlying cancer, i.e. metabolic complications (hypercalcemia, tumour lysis syndrome), pulmonary complications (airway obstruction, pleural effusion), urinary tract obstruction or haematologic complications (disseminated intravascular coagulation, hemophagocytic lymphohistiocytosis), pulmonary lymphangitic carcinomatosis, cardiac tamponade, tumoral bleeding and epilepsy related to brain metastasis; (ii) non-specific if the primary cause for ICU admission was a generic complication including infection, venous thrombo-embolism, non-tumoral bleeding, acute ischaemic events (myocardial infarction, stroke, mesenteric ischaemia, and limb ischaemia); (iii) adverse events distributed in drug-related side effects, based on intrinsic and extrinsic imputation, and procedural adverse events.

### Statistical analysis

Continuous variables were expressed as median (interquartile range) and categorical variables as counts (percentages) and were compared over time using Cuzick test and Chi-square test for trend, respectively.

In order to take into account the competitive risks and the time-dependent bias, the independent predictors of ICU death were addressed in a multivariate Cox cause-specific model, by performing a stepwise backward and forward variable selections based on Akaike information criteria [11, 12]. The model included variables that reached a p value of less than 0.20 in univariate analysis. Owing to the large number of missing data, the performance status was not entered into the model. Proportional hazard assumption was graphically checked and potential interactions were tested in the final model. DFLST was not included for short-term analysis because of self-fulfilling prophecy risk and to avoid immortality time bias. A similar analysis including DFLST was performed in ICU survivors to identify the determinants of one-year vital status.

All tests were two sided, and p values < 0.05 were considered statistically significant. All analyses were carried out using R 3.5.1 and R Studio (R foundation for Statistical Computing Vienna, Austria).

## Results

### Characteristics of ICU admissions

Between 2007 and 2018, 17,912 patients were admitted to our ICU (Additional file 1: Figure S1), of whom 1525 patients formed the cohort of interest of this study.

Patients’ characteristics are described in Tables 1 and 2. Median age was 67 years [59–75] with a majority of men. The two most frequent primary tumour sites were the gastro-intestinal tract (26.0%) and lung (24.9%). The number of ICU admissions increased by 76% between 2007–2008 and 2017–2018, mainly related to patients with lung and gastro-intestinal malignancies, and to a lesser extent with skin cancer. The proportion of patients with metastatic diseases increased from 48.6% in 2007–2008 to 60.2% in 2017–2018 (*p* = 0.004). The performance status prior to the acute complication could be accurately collected for 549 patients, and was severely impaired (stage 3 or 4) in 137 (25%) of them. The proportion of patients treated with antineoplastic treatments within the last 3 months increased from 38.5% in 2007–2008 to 53.3% in 2017–2018 (*p* = 0.001) along with a growing use of immunotherapy or targeted therapy (4.4% in 2007–2008 to 18.8% in 2017–2018, *p* < 0.001). Critical conditions were increasingly related to drug- or procedure-related adverse events, from 8.8% of ICU admissions in 2007–2008 to 16% in 2017–2018 (*p* = 0.01). Among 57 drug-related adverse events, 10 were related to targeted therapies and seven to immune checkpoint inhibitors. The crude severity of critical illness as assessed by the SOFA score at ICU admission did not change over time. Accordingly, further requirements for ventilatory or circulatory supports did not change either, although the use of renal replacement therapy substantially decreased.

**Table 1:** Changes in patients’ underlying characteristics between 2007 and 2018

Characteristics	2007–2008	2009–2010	2011–2012	2013–2014	2015–2016	2017–2018	p
	(*n* = 181)	(*n* = 230)	(*n* = 241)	(*n* = 263)	(*n* = 291)	(*n* = 319)	
Proportions of concurrent ICU admissions (%)	6.1	7.5	7.4	7.2	8.4	10.2	< 0.001
Age (years)	70 [59–78]	68 [59–76]	67 [60–75]	66 [57–75]	67 [59–74]	67 [60–74]	0.15
Male gender	126 (69.6)	143 (62.2)	152 (63.1)	159 (60.5)	163 (56)	201 (63)	0.87
Non-cancer comorbid conditions							
Hypertension	76 (42.0)	97 (42.2)	97 (40.2)	120 (45.6)	131 (45.0)	138 (43.3)	0.45
Diabetes	38 (21.0)	44 (19.1)	31 (12.9)	60 (22.8)	55 (18.9)	61 (19.1)	0.88
Cirrhosis	10 (5.5)	18 (7.8)	17 (7.1)	17 (6.5)	19 (6.5)	20 (6.3)	0.85
Chronic renal failure	20 (11.0)	20 (8.7)	18 (7.5)	29 (11.0)	28 (9.6)	22 (6.9)	0.34
Chronic heart failure	8 (4.4)	14 (6.1)	9 (3.7)	23 (8.7)	11 (3.8)	16 (5.0)	0.93
Type of cancer							< 0.001
Lung	50 (27.6)	55 (24.1)	50 (20.7)	60 (22.8)	69 (23.7)	95 (29.8)	
Gastrointestinal	25 (13.8)	44 (19.1)	72 (29.9)	90 (34.2)	86 (29.5)	80 (25.1)	
Urologic	51 (28.2)	63 (27.4)	48 (19.9)	47 (17.9)	47 (16.2)	58 (18.2)	
Breast	16 (8.8)	29 (12.7)	22 (9.1)	24 (9.1)	34 (11.7)	28 (8.8)	
Head and neck	12 (6.6)	11 (4.8)	12 (5.0)	5 (1.9)	7 (2.4)	7 (2.2)	
Gynaecologic	10 (5.5)	8 (3.5)	13 (5.4)	9 (3.4)	10 (3.4)	13 (4.1)	
Skin	1 (0.6)	3 (1.3)	2 (0.8)	3 (1.1)	10 (3.4)	7 (2.2)	
Others	16 (8.9)	17 (7.4)	22 (9.1)	25 (9.5)	28 (9.6)	31 (9.7)	
Time from diagnosis to ICU admission (days)	259 [43–722]	237 [43–833]	254 [61–967]	291 [99–1024]	267 [57–914]	264 [70–736]	0.23
Stage ^a^							0.004
Localized	53 (29.3)	66 (28.7)	65 (27.0)	58 (22.1)	70 (24.1)	60 (18.8)	
Advanced	34 (18.8)	55 (23.9)	50 (20.7)	46 (17.5)	62 (21.3)	67 (21.0)	
Metastatic	88 (48.6)	106 (46.1)	125 (51.9)	157 (59.7)	156 (53.6)	192 (60.2)	
Current cancer status							< 0.001
Newly diagnosed	62 (34.3)	72 (31.3)	71 (29.5)	58 (22.1)	96 (33.0)	82 (25.7)	
Partial remission	19 (10.5)	26 (11.3)	47 (19.5)	70 (26.6)	55 (18.9)	83 (26.0)	
Complete remission	26 (14.4)	48 (20.9)	37 (15.4)	37 (14.1)	36 (12.4)	36 (11.3)	
Progressive	65 (35.9)	80 (34.8)	85 (35.3)	94 (35.7)	102 (35.1)	114 (35.7)	
Recent oncological treatment (< 3 months)							
Antitumoral drug treatment	69 (38.5)	98 (42.8)	115 (47.7)	141 (53.6)	163 (56.0)	170 (53.3)	0.001
Immunotherapy and targeted therapy	8 (4.4)	18 (7.8)	17 (7.1)	35 (13.3)	37 (12.7)	60 (18.8)	< 0.001
First-line treatment	45 (25.0)	58 (25.2)	63 (26.1)	4 (28.1)	91 (31.3)	83 (26.0)	0.577
Radiotherapy	13 (7.2)	17 (7.4)	16 (6.7)	22 (8.4)	17 (5.8)	22 (6.9)	0.543
Surgery	30 (16.8)	38 (16.5)	35 (14.5)	37 (14.1)	25 (8.6)	47 (14.7)	0.184

^a^ Available for 1510 (99%) patients

Continuous variables are expressed as median (interquartile range) and categorical variables as counts (percentages)

**Table 2:** Changes in characteristics and outcomes of ICU admissions between 2007 and 2018

Characteristics	2007–2008	2009–2010	2011–2012	2013–2014	2015–2016	2017–2018	p
	(*n* = 181)	(*n* = 230)	(*n* = 241)	(*n* = 263)	(*n* = 291)	(*n* = 319)	
Features at ICU admission							
Time between hospital and ICU admissions (days)	0 [0–4]	0 [0–3]	0 [0–3]	0 [0–2]	0 [0–3]	0 [0–4]	0.625
SOFA score (points)	5 [4–9]	5 [4–8]	5 [4–8]	5 [4–8]	5 [4–7.50]	5 [4–9]	0.11
Leukopenia	4 (2.2)	9 (3.9)	15 (6.2)	13 (4.9)	15 (5.2)	17 (5.3)	0.05
Distribution of acute complications							
Non-specific	113 (62.4)	147 (63.9)	147 (61.0)	174 (66.2)	179 (61.5)	193 (60.5)	0.56
Infection	58 (32.0)	66 (28.7)	75 (31.1)	91 (34.6)	104 (35.7)	98 (30.7)	
Bleeding	6 (3.3)	10 (4.3)	9 (3.7)	17 (6.5)	18 (6.2)	19 (6.0)	
Ischaemic events	3 (1.7)	2 (0.9)	2 (0.8)	1 (0.4)	4 (1.4)	3 (0.9)	
Venous thrombo-embolism	4 (2.2)	8 (3.5)	7 (2.9)	9 (3.4)	6 (2.1)	11 (3.4)	
Miscellaneous	40 (22.1)	61 (26.5)	54 (22.4)	56 (21.3)	47 (16.2)	62 (19.4)	
Specific	52 (28.7)	62 (27.0)	71 (29.5)	65 (24.7)	84 (28.9)	75 (23.5)	0.26
Metabolic	3 (1.7)	9 (3.9)	9 (3.7)	7 (2.7)	8 (2.7)	5 (1.6)	
Respiratory	20 (11.0)	9 (3.9)	9 (3.7)	15 (5.7)	6 (2.1)	10 (3.1)	
Urinary tract obstruction	13 (7.2)	8 (3.5)	12 (5.0)	6 (2.3)	12 (4.1)	3 (0.9)	
Haematologic	0 (0.0)	2 (0.9)	0 (0.0)	0 (0.0)	2 (0.7)	0 (0.0)	
Others	16 (8.8)	34 (14.8)	41 (17.0)	37 (14.1)	58 (19.9)	57 (17.9)	
Adverse events	16 (8.8)	21 (9.1)	23 (9.5)	24 (9.1)	28 (9.6)	51 (16.0)	0.01
Drug-related adverse events	5 (2.8)	7 (3.0)	5 (2.1)	10 (3.8)	16 (5.5)	14 (4.4)	
Procedural adverse events	11 (6.1)	14 (6.1)	18 (7.5)	14 (5.3)	12 (4.1)	37 (11.6)	
Management during the ICU stay							
Organ failure supports							
Invasive mechanical ventilation	80 (44.2)	100 (43.5)	99 (41.1)	101 (38.4)	113 (38.8)	135 (42.3)	0.415
Non-invasive mechanical ventilation	29 (16.0)	20 (8.7)	21 (8.7)	28 (10.6)	23 (7.9)	42 (13.2)	0.72
Vasopressors/inotropes	74 (40.9)	79 (34.3)	81 (33.6)	75 (28.5)	96 (33.0)	107 (33.5)	0.14
Renal replacement therapy	48 (26.5)	44 (19.1)	43 (17.8)	39 (14.8)	50 (17.2)	35 (11.0)	< 0.001
Length of stay in the ICU (days)	3 [1–8]	3 [1–6]	2 [1–6]	2 [1–4]	3 [1–5]	3 [1–6]	0.91
Decision to forgo life-sustaining therapy	49 (27.1)	62 (27.0)	51 (21.2)	70 (26.6)	85 (29.2)	113 (35.4)	0.008
Outcomes							
ICU survival	126 (69.6)	176 (76.5)	203 (84.2)	203 (77.2)	232 (79.7)	240 (75.2)	0.407
Hospital survival^a^	83/168 (49.4)	112/203 (55.2)	136/224 (60.7)	151/250 (60.4)	176/281 (62.6)	166/298 (55.7)	0.75
One-year survival^b^	44/146 (30.1)	60/183 (32.8)	70/202 (34.7)	77/223 (34.5)	88/255 (34.5)	85/270 (31.5)	0.68

^a^ In-hospital survival status was available for 1424 patients

^b^ Survival status at one year was available for 1279 patients

Continuous variables are expressed as median (interquartile range) and categorical variables as counts (percentages)

### Outcomes and prognostic factors

**Fig. 1:**
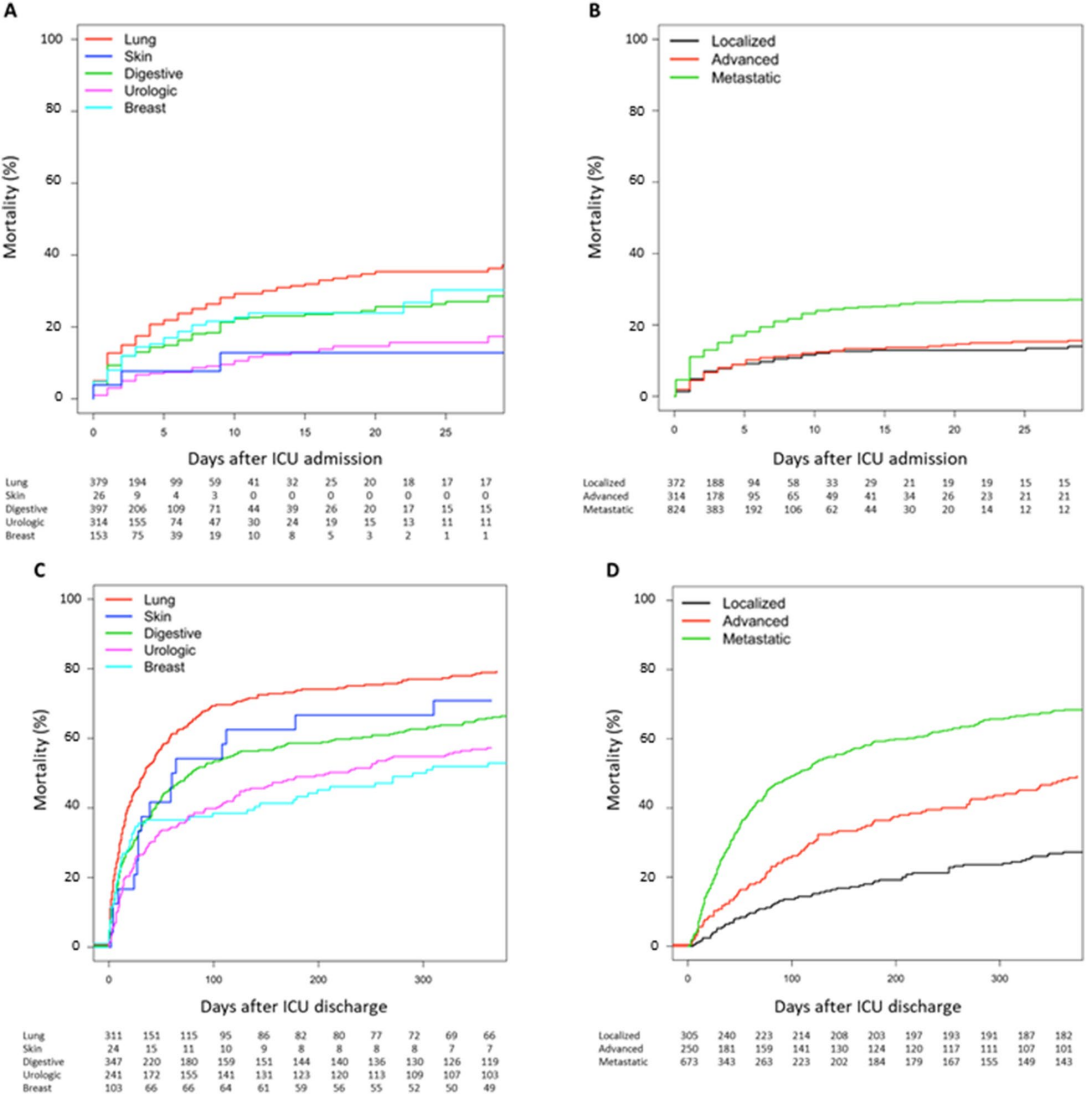
Short-term and one-year survival status. 28-day mortality was assessed from ICU admission according to the type (**A**) and stage (**B**) of cancer. One-year mortality was assessed in ICU-survivors according to the type (**C**) and stage (**D**) of cancer. The landmark is set at the time of ICU discharge. Patients with alternative types of solid tumours are not displayed in this figure

The ICU survival rate was 77.4%, without any significant changes over the study period. Among the 1279 patients with complete follow-up, the one-year survival rate was 33.2%. Short-term and long-term vital status, according to the type and stage of cancer, are displayed in Fig. 1. Among 137 patients with severely impaired performance status (stage 3 or 4), 84 (61%) and 2 (1.4%) survived the ICU stay and at one year, respectively. DFLST were increasingly taken during the ICU stay and/or at ICU discharge (35.4% in 2017–2018 vs. 27.1% in 2007–2008, *p* = 0.008). In-ICU and one-year mortality rates of patients with DFLST were 62.6% and 97.2%, respectively.

In multivariate analysis, independent factors associated with ICU mortality were metastatic disease (cause-specific hazard (CSH) 1.78 [1.38–2.30], *p* < 0.001), cancer in progression (CSH 1.62 [1.28–2.05], *p* < 0.001), need for invasive ventilation (CSH 3.73 [2.45–5.69], *p* < 0.001), non-invasive ventilation (CSH 2.87 [1.65–5.01], *p* < 0.001), inotropes/vasopressors (CSH 1.74 [1.29–2.34], *p* < 0.001) or renal replacement therapy (CSH 1.33 [1.03–1.70], *p* = 0.03), admission for specific complications (CSH 1.33 [1.04–1.72], *p* = 0.007) and SOFA score (CSH per point 1.02 [1.01–1.03], *p* < 0.001). Compared to patients with non-lung cancer, those with lung cancer had a worse prognosis in ICU (CSH 1.32 [1.04–1.66], *p* = 0.02) (Table 3).

**Table 3:** Determinants of ICU mortality

Characteristics	Survivors	Deceased	*p*	Multivariate analysis	
	(*n* = 1180)	(*n* = 345)		Cause-specific hazard [95% CI]	*p*
Age (years)	67 [59–75]	68 [60–74]	0.7		
Male gender	714 (60.5)	230 (66.7)	0.17		
ICU admission					
2007–2012	505 (39.1)	147 (43.6)	0.13		
2013–2018	675 (60.9)	198 (57.4)			
Type of cancer			< 0.001		
Lung	257 (21.8)	122 (35.4)			
Gastrointestinal	298 (25.3)	99 (28.7)			
Urologic	275 (23.3)	39 (11.3)			
Cutaneous	23 (2.0)	3 (0.9)			
Breast	123 (10.4)	30 (8.7)			
Lung cancer	257 (21.8)	122 (35.4)	< 0.001	1.32 [1.04–1.66]	0.02
Non-lung cancer	923 (78.2)	223 (64.6)		–	–
Stage			< 0.001		
Localized	314 (26.6)	58 (16.8)		–	–
Advanced	258 (21.9)	56 (16.2)		–	–
Metastatic	597 (50.6)	227 (65.8)		1.78 [1.38–2.30]	< 0.001
Status			< 0.001		
Newly diagnosed	314 (26.6)	105 (30.4)		–	–
Partial remission	259 (21.9)	41 (11.9)		–	–
Complete remission	182 (15.4)	38 (11.0)		–	–
In progression	384 (32.5)	156 (45.2)		1.62 [1.28–2.05]	< 0.001
Treatment in the past 3 months					
Chemotherapy	575 (48.7)	181 (52.5)	0.215	–	–
Surgery	183 (15.5)	29 (8.4)	0.003	0.68 [0.45–1.01]	0.05
Immunotherapy/targeted therapy	129 (10.9)	46 (13.3)	0.256	–	–
SOFA score	5 [4–7]	8 [5–12]	< 0.001	1.02 [1.01–1.03]^a^	< 0.001
Organ failure supports					
Invasive mechanical ventilation	338 (28.6)	290 (84.1)	< 0.001	3.73 [2.45–5.69]	< 0.001
Non-invasive mechanical ventilation	142 (12.0)	21 (6.1)	0.002	2.87 [1.65–5.01]	< 0.001
Inotropes/vasopressors	265 (22.5)	247 (71.6)	< 0.001	1.74 [1.29–2.34]	< 0.001
Renal replacement therapy	144 (12.2)	115 (33.3)	< 0.001	1.33 [1.03–1.70]	0.03
Admission patterns					
Non-specific complications	729 (61.8)	224 (64.9)	0.318	–	–
Specific complications	313 (26.5)	96 (27.8)	0.681	1.33 [1.04–1.72]	0.007
Adverse event complications	138 (11.7)	25 (7.2)	0.024	–	–
Drug-related adverse event	51 (4.3)	6 (1.7)	0.039		
Procedural adverse event	87 (7.4)	19 (5.5)	0.281		

^a^The CSH is related to 1-point increase in the SOFA score

We also addressed the determinants of one-year outcome in 934 ICU survivors (Table 4). One-year mortality was independently associated with lung cancer (CSH 1.44 [1.16–1.78], *p* < 0.001), metastatic disease (CSH 1.90 [1.53–2.37], *p* < 0.001), cancer in progression (CSH 1.39 [1.13–1.72], *p* = 0.002), admission for specific complications (CSH 1.75 [1.43–2.15], *p* < 0.001) and decision to forgo life-sustaining therapies (CSH 3.21 [2.54–4.06], *p* < 0.001).

**Table 4:** Determinants of one-year mortality in ICU-survivors

Characteristics	Alive	Deceased	*p*	Multivariate analysis	
	(*n* = 424)	(n = 510)		Cause-specific hazard [95% CI]	*p*
Age (years)	67 [59–74]	68 [60–76]	0.18	1.008 [1.007–1.012]	0.03
Male gender	248 (58.5)	297 (58.2)	0.4		
ICU admission					
2007–2012	174 (42.1)	210 (42.2)	0.54		
2013–2018	250 (58.1)	300 (58.8)			
Type of cancer			< 0.001		
Lung	65 (15.3)	139 (27.3)			
Gastrointestinal	116 (27.4)	141 (27.7)			
Urologic	103 (24.3)	109 (21.4)			
Cutaneous	7 (1.7)	14 (2.8)			
Breast	48 (11.3)	26 (5.1)			
Lung cancer	65 (15.3)	139 (27.3)	< 0.001	1.44 [1.16–1.78]	< 0.001
Non-lung cancer	359 (84.7)	371 (72.7)		–	–
Stage			< 0.001		
Localized	181 (42.7)	73 (14.3)		–	–
Advanced	99 (23.3)	104 (20.4)		–	–
Metastatic	141 (33.3)	329 (64.5)		1.90 [1.53–2.37]	< 0.001
Status			< 0.001		
Newly diagnosed	112 (26.4)	164 (32.2)		–	–
Partial remission	118 (27.8)	80 (15.7)		–	–
Complete remission	116 (27.4)	34 (6.7)		–	–
In progression	78 (18.4)	232 (45.5)		1.39 [1.13–1.72]	0.002
Treatment in the past 3 months					
Chemotherapy	172 (40.5)	272 (53.3)	< 0.001	–	–
Surgery	93 (21.9)	55 (10.8)	1.000	0.78 [0.57–1.05]	0.11
Immunotherapy/targeted therapy	32 (7.5)	72 (14.1)	0.002	–	–
SOFA score	5 [3.75–7]	5 [4–7]	0.379	1.03 [0.99–1.06] ^a^	0.05
Organ failure supports					
Invasive mechanical ventilation	121 (28.5)	140 (27.5)	0.519	–	–
Non-invasive mechanical ventilation	54 (12.7)	59 (11.6)	0.657	–	–
Inotropes/vasopressors	91 (21.5)	111 (21.8)	0.975	–	–
Renal replacement therapy	49 (11.6)	64 (12.5)	0.717	–	–
Admission patterns					
Non-specific complications	292 (68.9)	288 (56.5)	< 0.001	–	–
Specific complications	67 (15.8)	178 (34.9)	< 0.001	1.75 [1.43–2.15]	< 0.001
Adverse event complications	65 (15.3)	44 (8.6)	0.002	–	–
Drug-related adverse event	17 (4.0)	24 (4.7)	0.721	–	–
Procedural adverse event	48 (11.3)	20 (3.9)	< 0.001	–	–
Decision to forgo life-sustaining therapy	11 (2.6)	120 (23.5)	< 0.001	3.21 [2.54–4.06]	< 0.001

^a^The CSH is related to 1-point increase in the SOFA score

## Discussion

The current therapeutic revolution of cancer dramatically challenges the classical prognostic factors of several malignant diseases, but also reveals new patterns of life-threatening side effects, altogether likely to impact on indications for life-sustaining therapies. We report here significant changes in the features of ICU admissions in patients with solid malignancies, owing to the increasing prevalence of patients with advanced stages of diseases and the increased proportion of patients admitted for drug- or procedure-related adverse events. It is noteworthy that both short-term and one-year survival rates were not impaired by the increasing proportions of metastatic diseases over the study period. Moreover, the proportion of organ failure supports did not change over time, meaning that those supports were considered for patients with advanced malignancies. Only renal replacement therapy was less frequent at the end of the study period, likely related to recent changes in practices towards more delayed initiation [13]. Our results suggest that some malignancies formerly viewed as end-stage conditions are actually prone to prolonged survival after recovery from critical illnesses.

The increasing prevalence of patients with metastasis is associated with a growing proportion of patients under immunotherapy or targeted molecules. Patients with advanced diseases no more eligible to cytostatic chemotherapy (e.g. metastatic melanoma) may still remain liable to alternative treatments resulting in prolonged progression-free and overall survival. Considering the therapeutic options and the predicted lifespan of cancer patients is paramount to the decision-making process of life-sustaining therapies. However, one should keep in mind that data supporting our prognostic assessment are generally drawn from relatively fit and somewhat selected patients, with the natural bias of retaining the more optimistic results. Whether these findings apply to patients in the aftermath of critical illness is highly questionable, with respect to impaired nutritional, functional and cognitive status and persistent chronic organ dysfunctions. We strongly advocate a decision-making process where admission and limitation policies are based on close interactions between intensivists and oncologists to confront the theoretical prognosis to the clinical situation, to delineate the realistic short-term and long-term objectives of ICU admission and thereby to provide fair and accurate assessments of the expected benefits and harms of ICU admissions.

Our results are consistent with a recent meta-analysis that reported improved short-term prognosis in solid cancer patients admitted to ICU and with the study of Ostermann et al. [14, 15]. This lack of survival improvement in our study could be explained by significant changes in the oncological characteristics of ICU-admitted patients. In the present study, cancer patients sustained encouraging short-term and long-term survival rates, despite the increase in the proportion of patients at metastatic stage over the study period. This suggests that patients with advanced-stage diseases admitted in the ICU were still carefully selected. Determinants of ICU outcome were mostly related to the extent of organ failures, and also to the type and stage of malignancies which account for paramount factors in the decision-making process of therapeutic limitations. Both short-term and long-term prognoses are highly dependent on the case-mix of patients, and especially on the proportion of high-risk malignancies [16–18], and we herein pointed the particular burden of lung cancer amongst other malignancies [9]. Determinants of one-year outcome in ICU survivors were related to the stage of malignancies and to the type of complications that warranted ICU admission. Progression in underlying malignancy was linked to worse short- and long-term prognosis. It is well known that specific neoplastic complications, i.e. directly driven by cancer through compression or infiltration of anatomic structures and paraneoplastic syndromes, are associated with a worse prognosis, since poorly reversible despite aggressive chemotherapy [19, 20]. Some malignancies with actionable oncogenic mutations, including a subset of lung adenocarcinoma, are amenable to fast-acting targeted therapies such as tyrosine-kinase inhibitors [21, 22]. Some clinical conditions with rapidly threatening acute organ failures may even prompt the empirical institution of targeted drugs pending final molecular characterization. In contrast, the slow mechanism of action of immunotherapy, dependent on potent activation of anti-tumoral immunity, makes it irrelevant to achieve a fast reversal of specific organ failures. An important finding from this report is the increase in admissions related to drug and procedural adverse events, thereby highlighting the hazards related to emerging cancer treatments, most especially immunotherapy [23].

We acknowledge some limitations. This was a single-centre study, underlined by the development of a comprehensive oncology project in our hospital over the last two decades, and these results may not be fully transposable elsewhere. The collection of data was retrospective, likely reliable for most clinical features, but definitely less accurate for more subjective assessments not systematically collected at admission such as functional status, a relevant prognosis factor in this setting [24, 25]. We were able to collect the one-year vital status for the majority of patients (79.2%) discharged from the ICU, but we could not assess how the critical illness altered the optimal continuation of anticancer treatment, nor the quality of life [16, 26].

## Conclusion

Intensive care medicine is committed to support and accompany the progress in medicine. Advances in the management and the prognosis of solid malignancies have substantially modified the ICU admission patterns. Despite underlying advanced and often metastatic malignancies, three out of four patients survived the ICU stay and about one-third remained alive at one year. Such encouraging outcomes should help change the dismal perception of critically ill cancer patients. How critical illness may impact on the long-term prognosis of cancer remains to be investigated.

## Supplementary Information

**Additional file 1: Figure S1.** Flowchart.

## Data Availability

The datasets used and/or analysed during the current study are available from the corresponding author on reasonable request.

## Abbreviations

SOFA: Sequential Organ Failure Assessment
ICU: Intensive Care Unit
DFLST: Decisions to forgo life-sustaining therapies
CSH: Cause-specific hazard
