# Supplementary figures and images for: Patterns of ICU admissions and outcomes in patients with solid malignancies over the revolution of cancer treatment
Source: Ann Intensive Care. 2021 Dec 24;11:182. doi: 10.1186/s13613-021-00968-5 (PMC8709803; doi:10.1186/s13613-021-00968-5)

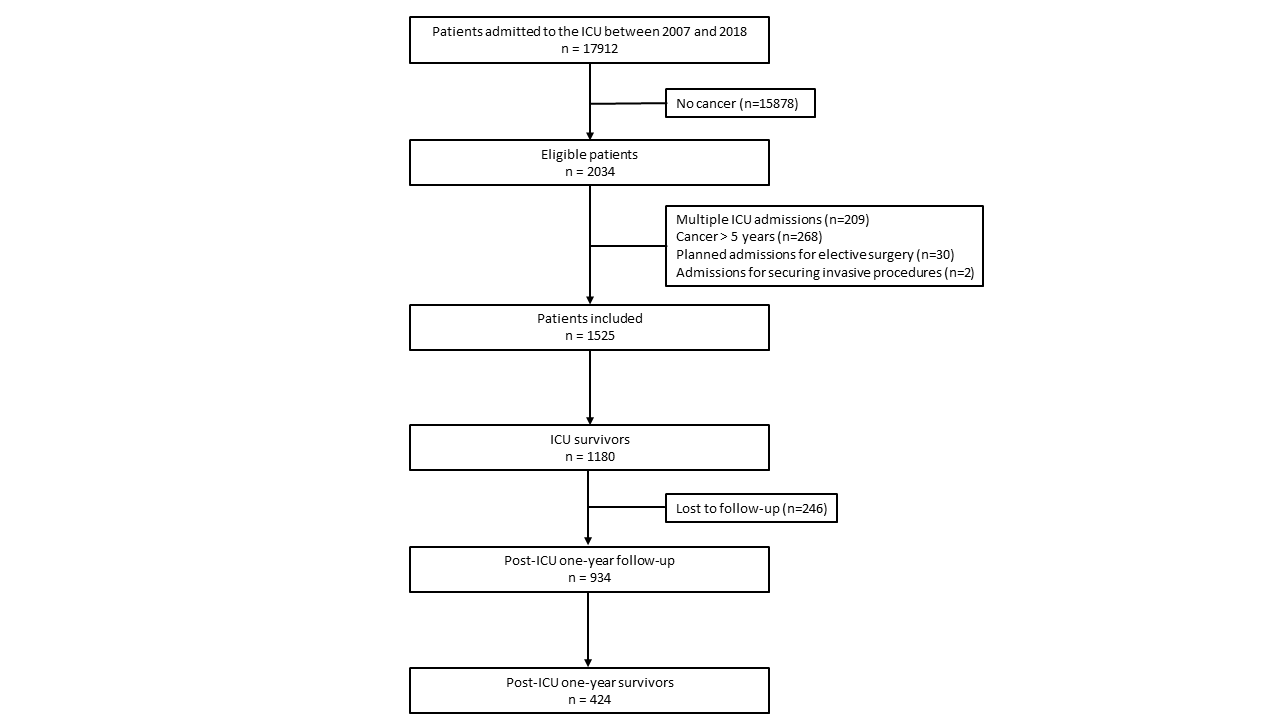

Supplement: Supplementary file 1 — Additional file 1: Figure S1. Flowchart. [file 13613_2021_968_MOESM1_ESM.tif]
